# The Effect of Two Amino acid Residue Substitutions via RNA Editing on Dark-operative Protochlorophyllide Oxidoreductase in the Black Pine Chloroplasts

**DOI:** 10.1038/s41598-017-02630-2

**Published:** 2017-05-24

**Authors:** Haruki Yamamoto, Junko Kusumi, Hisanori Yamakawa, Yuichi Fujita

**Affiliations:** 1 Graduate School of Bioagricultural Sciences, Nagoya University, Nagoya, 464-8601 Japan; 2 Department of Environmental Changes, Faculty of Social and Cultural Studies, Kyushu University, Fukuoka, 819-0395 Japan; 3 Department of Molecular and Cellular Biochemistry, Indiana University, IN, 47405-7003 USA

## Abstract

Dark-operative protochlorophyllide oxidoreductase (DPOR) is a key enzyme to produce chlorophyll in the dark. Among photosynthetic eukaryotes, all three subunits *chlL*, *chlN*, and *chlB* are encoded by plastid genomes. In some gymnosperms, two codons of *chlB* mRNA are changed by RNA editing to codons encoding evolutionarily conserved amino acid residues. However, the effect of these substitutions on DPOR activity remains unknown. We first prepared cyanobacterial ChlB variants with amino acid substitution(s) to mimic ChlB translated from pre-edited mRNA. Their activities were evaluated by measuring chlorophyll content of dark-grown transformants of a *chlB*-lacking mutant of the cyanobacterium *Leptolyngbya boryana* that was complemented with pre-edited mimic *chlB* variants. The chlorophyll content of the transformant cells expressing the ChlB variant from the fully pre-edited mRNA was only one-fourth of the control cells. Co-purification experiments of ChlB with Strep-ChlN suggested that a stable complex with ChlN is greatly impaired in the substituted ChlB variant. We then confirmed that RNA editing efficiency was markedly greater in the dark than in the light in cotyledons of the black pine *Pinus thunbergii*. These results indicate that RNA editing on *chlB* mRNA is important to maintain appropriate DPOR activity in black pine chloroplasts.

## Introduction

**Figure 1:**
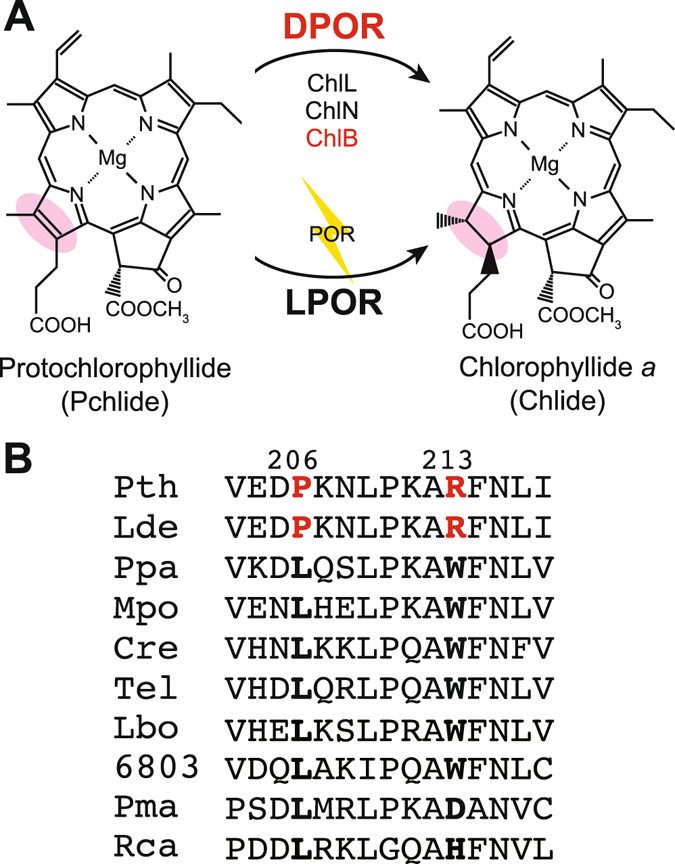
Protochlorophyllide reduction (**A**). Protochlorophyllide (Pchlide) is reduced by two structurally unrelated enzymes; LPOR and DPOR. DPOR is a nitrogenase-like oxygen-labile enzyme consisting of the three subunits ChlL, ChlN, and ChlB and serves as a key enzyme for Chl biosynthesis in the dark. (**B**) Partial amino acid sequences of chloroplast DNA encoded ChlB from *P. thunbergii* (Pth), *L. decidua* (Lde), *P. patens* (Ppa), the liverwort *Marchantia polymorpha* (Mpo), the green alga *Chlamydomonas reinhardtii* (Cre), *Thermosynechococcus elongatus* (Tel), *Leptolyngbya boryana* (Lbo), *Synechocystis* sp. PCC 6803 (6803), *Prochlorococcus marinus* SS120 (Pma), and the purple bacterium *Rhodobacter capsulatus* (Rca). RNA editing derived from amino acid substitutions is shown in bold, and the two amino acid residues from the pre-edited transcript in *P. thunbergii* and *L. decidua* are shown in red. The numbers 206 and 213 are from *P. thunbergii* ChlB.

Chlorophyll *a* (Chl) is an essential tetrapyrrole pigment in photosynthesis that is synthesized from glutamate through a complex pathway consisting of at least 15 enzymes^1–3^. Intermediates of Chl produce reactive oxygen species upon exposure to light. Consequently, Chl biosynthesis is tightly regulated in response to a variety of environmental factors, including light and redox states^4^. In angiosperms, light is used for photosynthesis and in addition is also a major environmental signal that regulates Chl biosynthesis. In these plants, light is required for reduction of protochlorophyllide (Pchlide) to chlorophyllide *a* by the enzyme light-dependent Pchlide oxidoreductase (LPOR). LPOR is the sole Pchlide reductase in angiosperms so seedlings grown in the dark are etiolated (lack Chl). Many angiosperms studies on Chl biosynthesis have focused on LPOR, leading to the discovery of the regulatory protein FLU^5^. Photosynthetic organisms that have a longer evolutionary history, such as gymnosperms, also have an alternative Pchlide reduction system called dark-operative Pchlide oxidoreductase (DPOR) that functions irrespective of light^6–9^. Most gymnosperm seedlings have both LPOR and DPOR allowing these plants to produce Chl even in the dark (Fig. 1A)^10, 11^.

Even though DPOR and LPOR catalyze the same stereospecific reduction of the C17=C18 double bond of Pchlide, these enzymes are structurally and evolutionarily unrelated^9^. LPOR is a nuclear-encoded protein that belongs to the short-chain dehydrogenase/reductase (SDR) superfamily, which consists of single polypeptide enzymes that use NADPH as a cofactor to catalyze a variety of redox reactions^12^. In contrast, DPOR is a nitrogenase-like enzyme encoded by three genes, *chlL*, *chlN* and *chlB*. The three gene products form two separable components, L protein (2ChlL dimer) and NB protein (2ChlN–2ChlB heterotetramer)^8^. The L protein transfers electrons to the NB protein coupled with ATP hydrolysis^13^, and the NB protein plays a role as the catalytic component of DPOR. The NB protein binds Pchlide, and the C17=C18 double bond of Pchlide is reduced by electrons transferred from the NB cluster (a [4Fe-4S] cluster). The NB cluster held by three Cys residues from ChlN and one Asp residue from ChlB is placed at the interface between ChlN and ChlB^14^. Thus, the complex formation between ChlN and ChlB is crucial for the catalytic activity of DPOR.

The *por* gene encoding LPOR is distributed throughout oxygenic photosynthetic organisms, including cyanobacteria. The three genes, *chlL*, *chlN* and *chlB*, for DPOR are present in photosynthetic bacteria (denoted as *bchL*, *bchN* and *bchB*, respectively), cyanobacteria, green algae, bryophytes, pteridophytes, and gymnosperms. In photosynthetic eukaryotes, the three genes are encoded in chloroplast genomes. However, the three genes coding for subunits of DPOR are absent in angiosperms and various eukaryotic algae^15^. Nonetheless, the physiological significance of the presence of both LPOR and DPOR in various photosynthetic organisms remains largely unknown, although light and oxygen appear to be key factors in their functional differentiation in cyanobacteria^16^ and it has been reported that DPOR is required for Chl production under short-day conditions in the liverwort *Marchantia polymorpha* ^17^.

RNA editing is a post-transcriptional modification that introduces changes in RNA sequences^18^. RNA editing in plant mitochondria and plastids modifies various codons to encode different amino acid residues as well as a few cases where initiation and stop codons are generated^19, 20^. In the black pine *Pinus thunbergii*, *chlN* and *chlB* in the chloroplast genome have been reported to have one and two RNA editing sites, respectively^11, 21, 22^. The site in *chlN* undergoes the conversion from the codon CCU (Pro285) to UCU (Ser), and the two sites in *chlB* undergo the conversion from codons CCG (Pro206) and CGG (Arg213) to CUG (Leu) and UGG (Trp), respectively. These three amino acids in ChlN and ChlB are highly conserved among many oxygenic phototrophs^23^ (Fig. 1B). The two C-to-U changes in *chlB* have also been found in the larch *Larix decidua* by Demko *et al*.^11^ who showed that the RNA editing efficiency of *chlB* mRNA correlates with the accumulation of Chl in the dark suggesting that RNA editing in *chlB* affects Chl biosynthesis in these gymnosperms. However, no direct causal links among these amino acid substitutions and DPOR activity has been identified, in part, because DPOR is extremely sensitive to oxygen and can be assayed only under anaerobic conditions *in vitro* ^16, 24, 25^.

To evaluate DPOR activity *in vivo*, we developed a complementation system in the cyanobacterium *Leptolyngbya boryana* ^24, 25^. In this system, a cyanobacterial *chlB* mutant, which lacks the ability to produce Chl in the dark, is used to express an exogenous *chlB* query gene. DPOR activity of the query gene is then evaluated by measuring the Chl content in transformant cells grown in the dark. In a previous study, we used this system to successfully evaluate chloroplast DNA-encoded DPOR activity from the moss *Physcomitrella patens* ^24^. In the present study, we focused on the activity of DPOR using cyanobacterial ChlB subunit variants carrying amino acid substitutions that correspond to those of pre-edited *chlB* mRNA in *P. thunbergii*. In addition, we analyzed changes in the RNA editing efficiency in *chlB* mRNA in response to light conditions in seedlings of *P. thunbergii*. These results suggest that RNA editing in *chlB* serves as a regulatory system for DPOR activity in response to environmental light conditions in black pine chloroplasts.

## Results

### Pre-edited mimic variants of cyanobacterial ChlB

Comparisons of ChlB amino acid sequences among various photosynthetic organisms that lack RNA editing processes indicated that the two amino acid residues in black pine ChlB that are generated by RNA editing (Leu206 and Trp213) are highly conserved among plants and cyanobacteria (Fig. 1B). The only significant divergence occurs at the second site that diverges in a group of marine cyanobacteria (Asp) and in proteobacteria (His) (Fig. 1B, Pma and Rca)^23^. To investigate whether these two amino acid residues are functionally important for DPOR activity, and to evaluate the activity of the edited and pre-edited forms of ChlB, we tried to express *chlB* together with *chlN* encoding the other subunit of the NB protein of DPOR from *P. thunbergii* in *Escherichia coli*. However, all portions of the expressed protein were recovered in the insoluble fraction of *E. coli*. We also tried to evaluate the activity of edited and pre-edited ChlB proteins *in vivo* using the *in vivo* complementation system of the cyanobacterium *L. boryana* ^24, 25^. However, Chl biosynthesis in the dark was not restored even with the edited ChlB from *P. thunbergii*, owing to very low expression of the black pine ChlN and ChlB proteins and probable incompatibility between the cyanobacterial L protein and the black pine NB protein.

**Figure 2:**
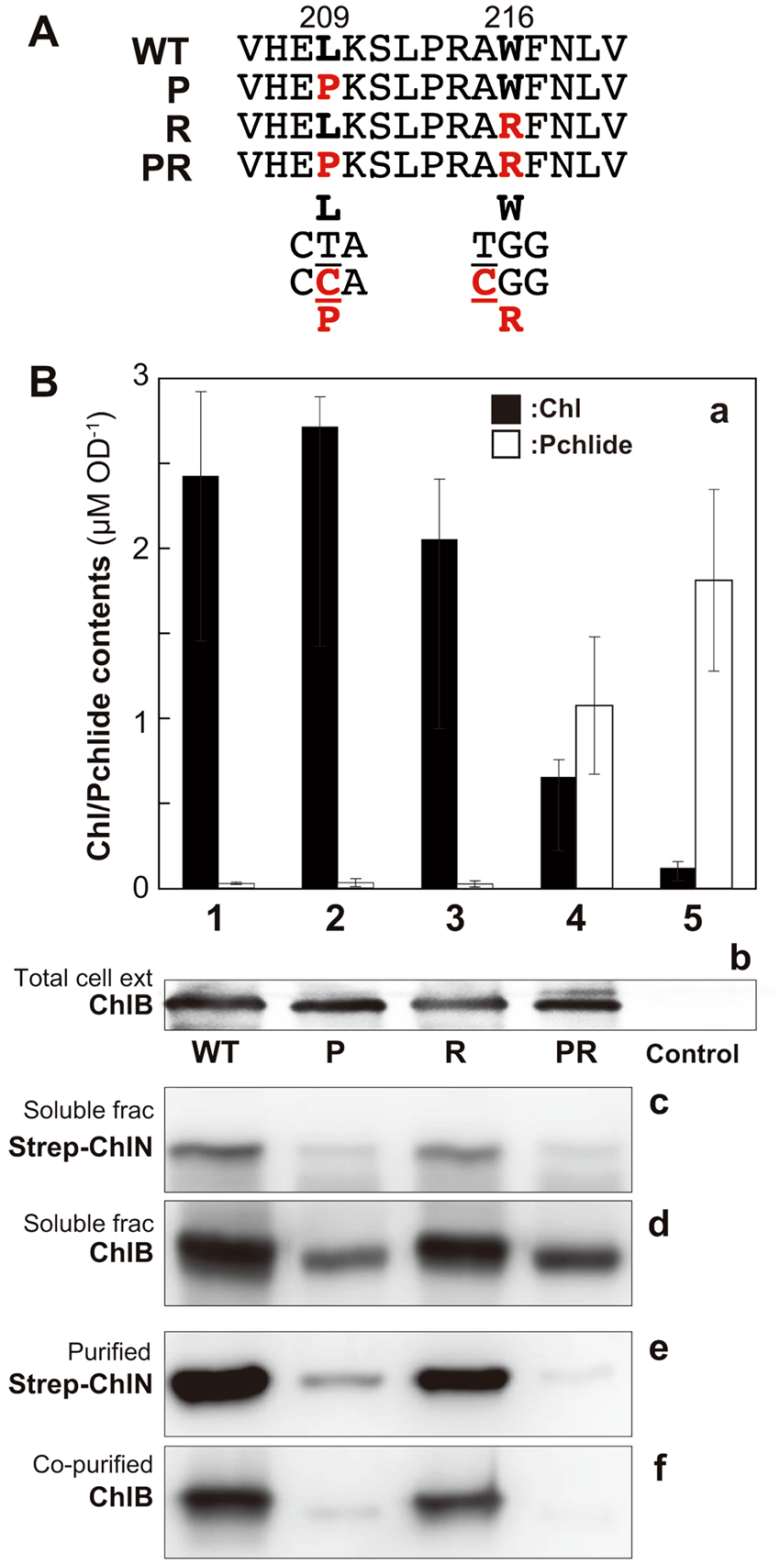
Effects of pre-edited mimic substitutions of ChlB in cyanobacterial cells. (**A**) The partial ChlB amino acid sequence from *L. boryana* (WT) and the pre-edited mimic sequences from pHBNB2_P (P), pHBNB2_R (R), and pHBNB2_PR (PR) are shown. The two codons (two T-to-C substitutions shown in red) were altered in the three plasmids. (**B**) (a) Chl and Pchlide contents of dark-grown transformants of YFB14 harboring the shuttle vectors pHBNB2 (WT, lane 1), pHBNB2_P (P, lane 2), pHBNB2_R (R, lane 3), pHBNB2_PR (PR, lane 4), and empty vector (Control, lane 5) (b) The presence of ChlB was confirmed by Western blot analysis of total cell extract of transformant cells using the anti-ChlB antiserum. (c, d) Strep-ChlN (c) and ChlB (d) in the soluble fractions were detected by anti-ChlN and anti-ChlB antisera, respectively. (e, f) Affinity purified proteins from these crude extracts were detected by anti-ChlN (e) and anti-ChlB (f) antisera, respectively.

Given that the Leu and Trp residues present in *P. thunbergii* ChlB are also conserved in ChlB from the cyanobacterium *L. boryana* (Figs 1B and 2A), we subsequently decided to evaluate the effects of Leu209 (CTA) to Pro209 (CCA) and Trp216 (TGG) to Arg216 (CGG) amino acid substitutions on DPOR activity in this cyanobacterial species. For *in vivo* analysis, we expressed ChlB variants bearing pre-edited codons Pro209 (ChlB_P) and Arg216 (ChlB_R) as well as a doubly pre-edited variant (ChlB_PR) bearing both pre-edited codons of Pro209 and Arg216 (Fig. 2A). Shuttle vectors carrying pre-edited mimic *chlB* variants with *strep*-*chlN* as an artificial operon *strep*-*chlN*–*chlB* as previously reported^24, 25^ were introduced into the *chlB*-lacking *L. boryana* mutant YFB14^26^. These transformants, YFB14/NB2, YFB14/NB2_P, YFB14/NB2_R, and YFB14/NB2_PR express the wild-type ChlB, ChlB_P, ChlB_R, and ChlB_PR variants respectively, as well as Strep-ChlN.

In YFB14, Chl synthesis in the dark is arrested at the Pchlide reduction step caused by a disruption of *chlB* ^26^. The Chl content of YFB14/NB2_P and YFB14/NB2_R cells grown in the dark was almost identical to that observed in the positive control YFB14/NB2 indicating that these mutations are functionally active (Fig. 2B,a). In contrast, the Chl content of YFB14/NB2_PR cells grown in the dark was significantly lower than that of the positive control. In addition, while only traces of Pchlide were detected in YFB14/NB, YFB14/NB2_P, and YFB14/NB2_R cells, the YFB14/NB2_PR cells contained markedly higher concentrations of Pchlide, reaching approximately 60% of that of the negative control (YFB14 with an empty pPBH202 vector^25^) (Fig. 2B,a). The Pchlide accumulations were also well correlated with decreased Chl contents. The contents of ChlB proteins in transformant cells were subsequently assessed by Western blot analysis (Fig. 2B,b). In all three YFB14/NB2 mutant variants, ChlB proteins were present at levels comparable to the amount of wild type ChlB present in the control strain YFB14/NB2, suggesting that only the PR mutant of ChlB is functionally impaired as a DPOR subunit in cyanobacterial cells.

### Co-purification of ChlB variants with Strep-ChlN

We checked the amount of ChlB in soluble fractions and observed that ChlB_R was present at same level as WT ChlB. This is contrasted by ChlB_P and ChlB_PR variants that were present at less than half the wild type level (Fig. 2B,d). This indicates that the Pro substitution reduced solubility of ChlB. In addition, the amount of Strep-ChlN, which does not have any amino acid substitutions, also largely decreased in the soluble fractions that contained ChlB_P and ChlB_PR variants (Fig. 2B,c).

We also addressed whether ChlB variants can form a stable complex with ChlN by performing a co-purification assay. For this assay, ChlB was co-purified with Strep-ChlN from cyanobacterial soluble extracts using a Strep-tag affinity column and the amount of co-purified ChlB was evaluated by Western blot analysis (Fig. 2B,f). This analysis showed that ChlB was indeed co-purified with Strep-ChlN from extracts that expressed ChlB_R as well as from extracts that expressed WT ChlB. However, the yield of Strep-ChlN from extracts that expressed ChlB_P was very low. Interestingly, equivalent low amounts of ChlB_P was co-purified with these low yields of Strep-ChlN. Little amount of Strep-ChlN was also obtained from extracts that expressed ChlB_PR. However in these extracts, no detectable ChlB_PR was observed (Fig. 2B,e,f).

**Figure 3:**
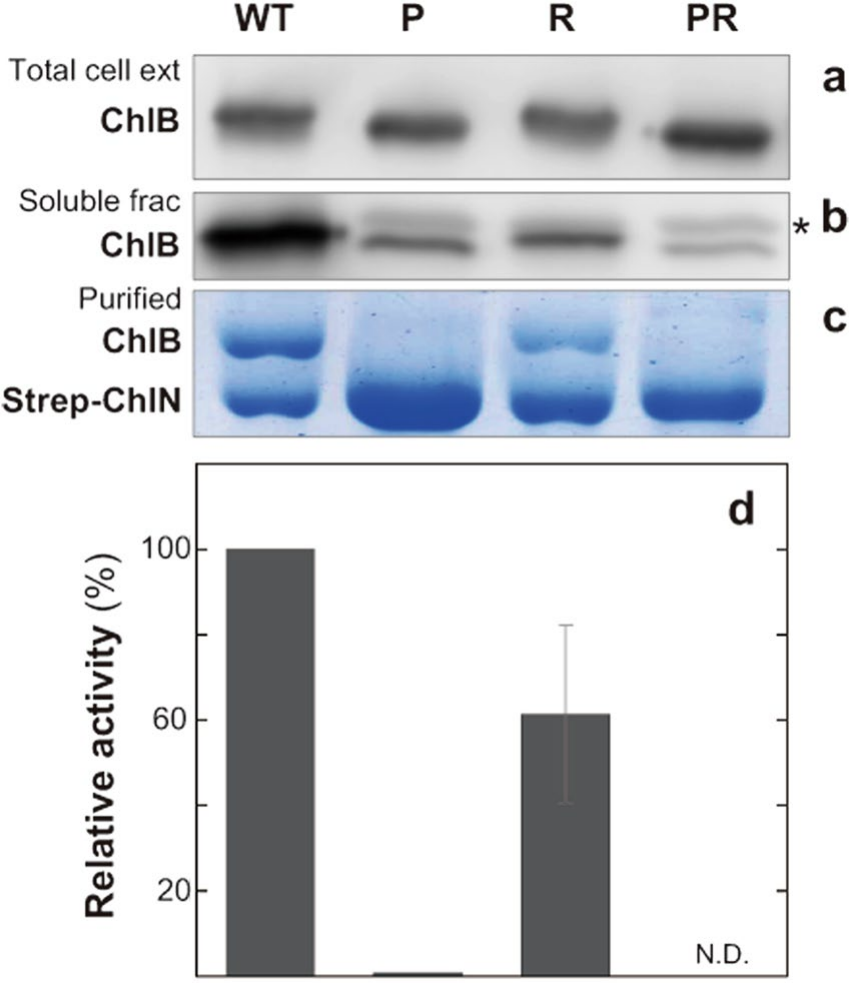
Effects of pre-edited mimic substitutions of ChlB expressed in *E. coli*. Purified NB protein with pre-edited mimic ChlB variants from *E. coli*. Western blot analysis of total extracts (a, 0.1 µg per lane) and soluble fractions (b, 1.25 µg per lane) of *E. coli* using the anti-ChlB antiserum, and SDS-PAGE profiles of affinity-purified NB protein variants (c, 3.0 µg per lane); Proteins were stained using Coomassie Brilliant Blue (c). A nonspecific signal detected just above the ChlB signal in the soluble fractions is shown by an asterisk (b). Relative DPOR activity was measured using Strep-purified proteins with ChlL (d). The 100% activity of the NB protein was 22.0 nmol min^−1^ mg_protein_^−1^. N.D. denotes “not detected”.

We also used an *E. coli* expression system to assay the interaction between the cyanobacterial ChlN and ChlB in heterologous cells (Fig. 3). In this *E. coli* expression assay, the amount of ChlB variants in the total cell free crude extract was about the same as observed with WT ChlB (Fig. 3,a). However, when assaying amount in a clarified soluble fraction there was significantly less soluble ChlB_PR than observed with the ChlB_R and ChlB_P variants (Fig. 3,b). In spite of the decreased amount of soluble ChlB proteins, a significant amount of ChlB was co-purified with Strep-ChlN from the extracts that expressed ChlB_R variant as well as WT control (Fig. 3,c). However, quantitation of the yields of Strep-ChlN and ChlB showed that while WT ChlB formed a stable ~1:1 interaction with Strep-ChlN, ChlB_R co-purified with Strep-ChlN at ~60% lower amounts. The observed ~60% reduction in co-purification of ChlB_R is similar to the observed ~60% reduction in relative activity (Fig. 3,d). On the other hand, ChlB_P and ChlB_PR both showed no detectable co-purification with Strep-ChlN (Fig. 3,c), which was consistent with no detectable activity of these proteins (Fig. 3,d). These results with *E. coli* extracts indicate that the formation of stable ChlN-ChlB complex is indeed inhibited by the Pro substitution with the Arg substitution enhancing this inhibitory effect.

### RNA editing of chlB transcripts in pine seedlings

**Figure 4:**
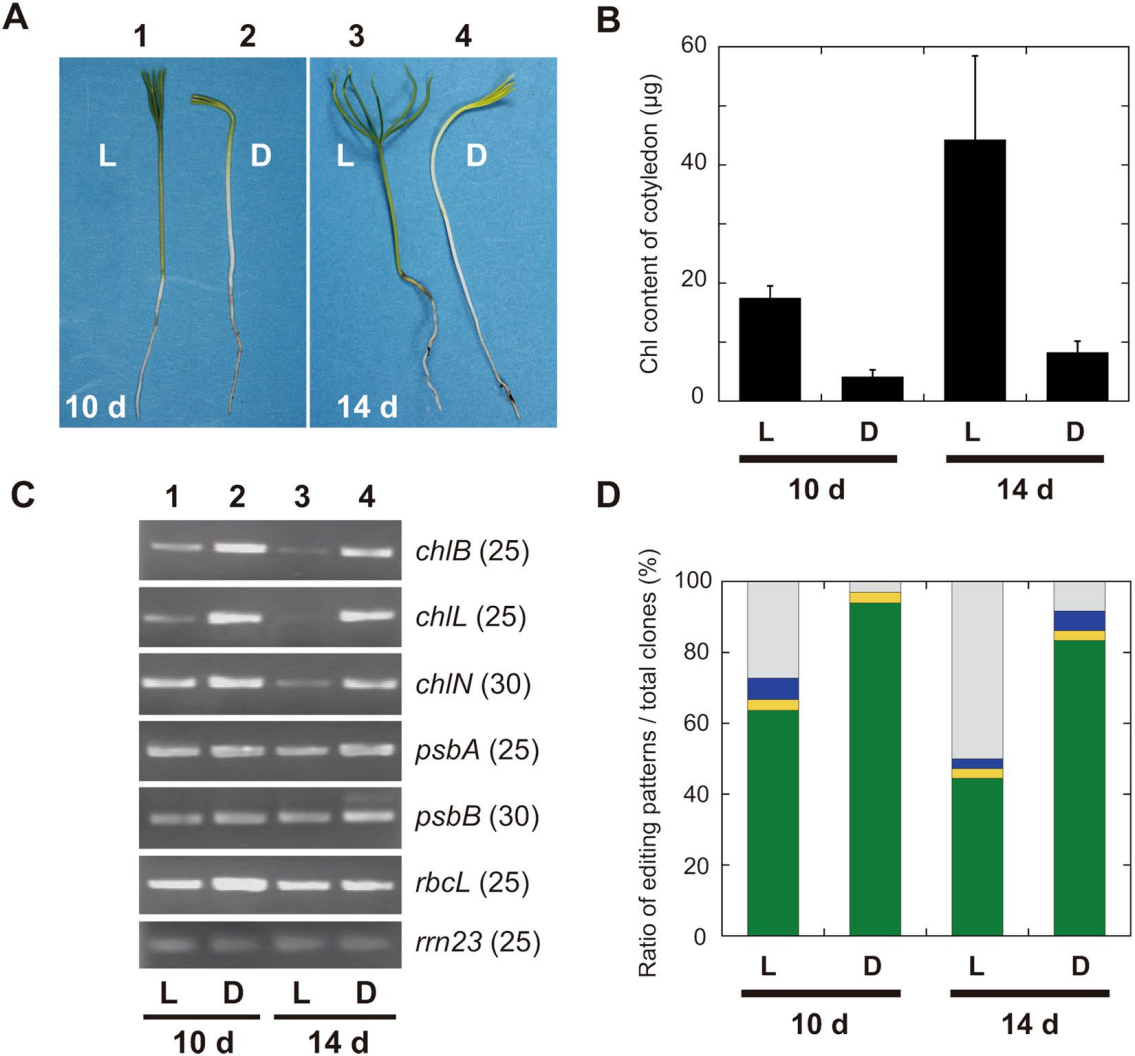
Efficiency of RNA editing of *chlB* mRNA in cotyledons of black pine (**A**). Seedlings of the black pine *P. thunbergii* were grown for 10 and 14 days under light (L) and dark (D) conditions (Scale bars = 10 mm). (**B**) Chl (including Chl *a* and Chl *b*) content (µg cotyledon^−1^) in the cotyledons at 10 and 14 days under light (L) and dark (D) conditions. (**C**) Semi-quantification of the transcripts of several chloroplast genes in cotyledons of seedlings grown in light (lanes 1 and 3) and dark (lanes 2 and 4) for 10 (lanes 1 and 2) and 14 days (lanes 3 and 4). Three *chlB*, *chlL*, and *chlN* genes encode the DPOR subunits. The *psbA*, *psbB*, and *rbcL* genes were treated as genes representative of photosynthesis in chloroplast genomes. *rrn23* was used as an internal standard. Cycle numbers are shown in parentheses. (**D**) The RNA editing efficiency of *chlB* transcript is expressed as the ratio of the number of clones with edited codons to the total number of examined clones. Ratios among clones (Supplementary Table S1) are color-coded; both edited codons (CTA/TGG), green; singly edited codons (CCA/TGG, CTA/CGG), yellow and blue, respectively; and pre-edited codons (CCA/CGG), light gray.

Black pine (*P. thunbergii*) seedlings were cultivated under continuous light or dark conditions for 10 and 14 days (Fig. 4A). The dark cultivated black pine seedlings produced Chl as indicated by a yellow–green color (Fig. 4A) with Chl content assays showing that the dark-grown seedlings produced 20%–25% of the amount of Chl observed with light-grown seedlings (Fig. 4B). To investigate the correlation between light-independent Chl production and DPOR gene expression, transcript levels of DPOR subunits were semi-quantified using reverse transcriptase (RT)-PCR (Fig. 4C). The transcript levels of *psbA*, *psbB*, and *rbcL*, which are encoded by the chloroplast genome, were almost constant under both light and dark conditions. This result is consistent with a previous report^27^. However, the transcript levels of all three DPOR genes were markedly higher in dark-grown seedlings than those in light-grown seedlings. This tendency was more pronounced after 14 days, with transcript levels in light-grown seedlings markedly decreasing from 10 days to 14 days. This result suggests that the expression of three DPOR genes is down-regulated in the light and/or up-regulated in the dark.

Two codons CCA (Pro206) and CGG (Arg213) of chloroplast DNA-encoded *chlB* are post-transcriptionally changed to CUA (Leu206) and UGG (Trp213) by RNA editing^11^. To determine the RNA editing efficiency of *chlB* mRNA, partial *chlB* fragments covering the two editing sites were amplified from cDNA samples using PCR and cloned into the vector. The nucleotide sequences of these *chlB* fragments were determined in more than 30 clones for each condition, and the RNA editing efficiency was estimated as the ratio of the number of edited clones (sequenced as CTA and TGG) to the total number of clones (Fig. 4D, Supplementary Table S1). In dark-grown seedlings, over 80%–90% of *chlB* transcripts were correctly edited in both codons while singly edited clones (CTA/CGG or CCA/TGG) present less than 10% of the clones. This result suggested that the two RNA editing reactions are tightly coupled or proceed very rapidly. In addition, the total numbers of each singly edited codon were almost identical, suggesting no site preference in the two editing sites. In contrast to the dark-grown seedlings, only 40%–60% of cDNA clones were edited at both sites in light-grown seedlings. The RNA editing efficiency for both sites in 10-day seedlings was slightly higher than that in 14-day seedlings grown in the dark, whereas that in 10-day light-grown seedlings was approximately 60% and decreased to approximately 40% in 14-day light-grown seedlings. These results indicate that light not only suppresses the transcription of DPOR genes but also reduces the efficiency of RNA editing of *chlB* mRNA.

## Discussion

In this study, we showed that a cyanobacterial ChlB variant (ChlB_PR) that mimics pre-edited plastid ChlB significantly decreases DPOR activity relative to wild-type ChlB. We also showed that the transcript levels of three plastid genes coding for DPOR subunits are up- and down-regulated in dark and light conditions, respectively, in seedlings of *P. thunbergii*. In addition, the RNA editing efficiency of *chlB* transcripts correlated well with transcript levels, being markedly higher in dark-grown than in light-grown seedlings. If the activity observed in the cyanobacterial ChlB variant is applicable to black pine chloroplast ChlB then this suggests that DPOR activity is also regulated at the post-transcriptional stage via RNA editing.

The single Pro substitution on the cyanobacterial ChlB (ChlB_P) at the same position as that which occurs by RNA editing of the eukaryotic homologs has a negative effect on formation of a complex with ChlN. In addition, this mutation also causes a decrease in solubility. These negative effects are also manifest by expression in *E. coli* cells in which no complex formation with ChlN was observed (Fig. 3,c). Interestingly, ChlB_P did complement Chl biosynthesis in *chlB* deletion strain (YFB14/NB2_P; Fig. 2B,a) indicating that this mutation can form enough of a functional NB complex in cyanobacterial cells to maintain Chl synthesize in the dark. These results also indicate that cyanobacterial cells may contain a stabilizing factor(s), such as Pchlide that allows the ChlB Pro substitution to form a NB complex. The additional Arg substitution (ChlB_PR) caused more severe effect on complex formation with ChlN with this negative effect observed even in cyanobacterial cells.

Interestingly, a significant decrease in the amount of ChlN was observed in YFB14/NB2_P and YFB14/NB2_PR strains that express the Pro substituted ChlB variants (Fig. 2B,c). Given that the Pro substitution affects formation of a stable NB protein complex, it is possible that a large part of ChlN may exist as a monomer in YFB14/NB2_P and YFB14/NB2_PR and that monomeric ChlN is unstable. Previous work on nitrogenase reported that NifD (corresponding to ChlN on DPOR) was rapidly degraded and accumulated as an insoluble aggregate without its partner NifK (corresponding to ChlB), while NifK was stable alone and formed homo tetramer^28^. Taken together, the results in *L. boryana* and *E. coli* suggest that the Pro substitution affects NB complex formation and the drastic decrease of DPOR activity observed with the pre-editing mimic ChlB variant (ChlB_PR) is caused by defect in complex formation with ChlN.

**Figure 5:**
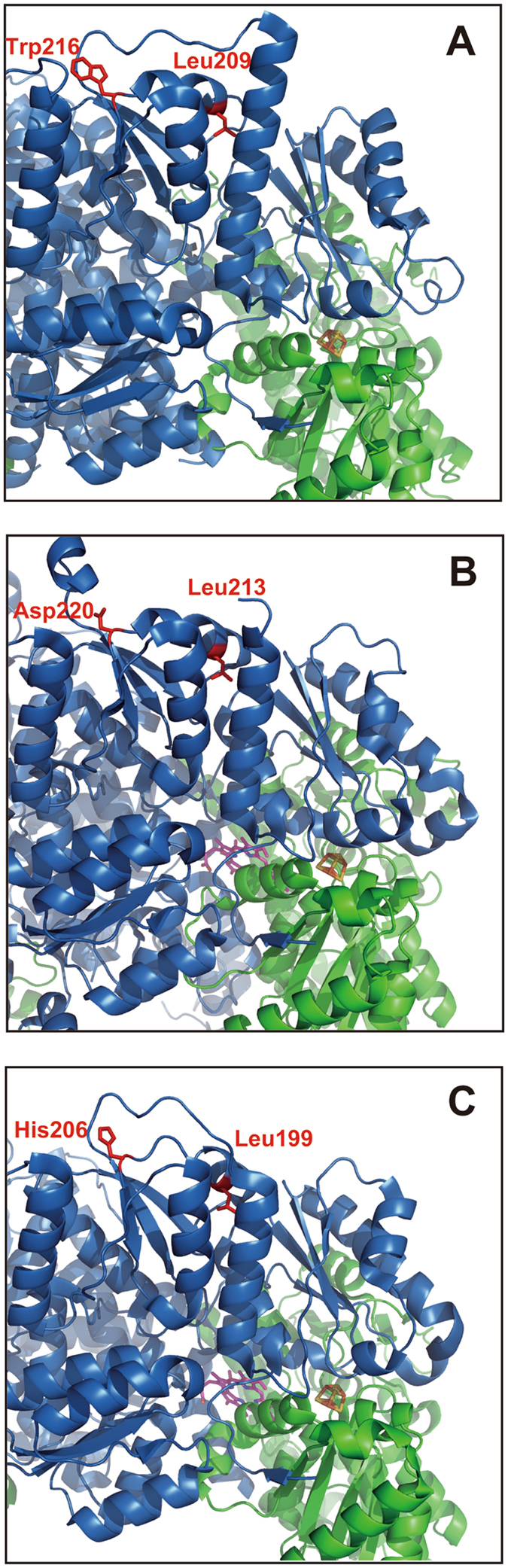
Spatial arrangement of the two editing amino acid residues corresponding to the edited codons in the crystal structures of NB proteins from three photosynthetic organisms. Close-up view of the two amino acid residues corresponding to editing sites in crystal structures of NB proteins from *T. elongatus* (**A**; PDB code 2XDQ), *P. marinus* (**B**; PDB code 2YNM), and *R. capsulatus* (**C**; PDB code 4AEK); ChlN/BchN and ChlB/BchB subunits are colored green and blue, respectively. The two amino acid residues corresponding to the editing sites are shown in red. The [4Fe-4S] clusters shown in a CPK model (yellow and orange) and Pchlide molecules are shown as stick models (pink). The crystal structure from *T. elongatus* is of the Pchlide-free form.

Crystal structures of the DPOR complex of L protein and NB protein from *Prochlorococcus marinus* has been recently reported as well as crystal structures of NB protein from *Rhodobacter capsulatus* and *Thermosynechococcus elongatus* ^14, 29, 30^. Although sequence similarity among these three ChlB is not high (the most distant pair: about 35% between *T. elongatus* and *P. marinus*), the overall structure of the NB protein is conserved very well. The spatial arrangement of these two edited amino acid residues, Leu and Trp (Asp/His), is almost identical in the structures of the three NB proteins (Fig. 5). Given that black pine ChlB shows high similarity to cyanobacterial ChlB (68% to *T. elongatus* and 65% to *L. boryana*), the observed effects of the PR substitution in *L. boryana* ChlB likely reflect the actual effects of amino acid substitutions by RNA editing in *P. thunbergii*.

The first editing site (Leu209 in *T. elongates* ChlB, Fig. 5A; Leu213 in *P. marinus* ChlB, Fig. 5B; and Leu199 in *R. capsulatus* BchB, Fig. 5C) is commonly present as a short α-helix consisting of nine amino acid residues that may stabilize the adjacent two α-helices via a hydrophobic interaction. The second editing site (Trp216 of the cyanobacterial ChlB, Fig. 5A), which is not conserved in *R. capsulatus* (His206, Fig. 5C) or *P. marinus* (Asp220, Fig. 5B), is commonly located in a very short loop connecting the α-helix that contains the first editing site and the short β-sheet. Common spatial positions of two residues among the three crystal structures are distal from the catalytic Pchlide-binding cavity, [4Fe-4S] cluster, and subunit-interaction region between ChlN/BchN and ChlB/BchB (Fig. 5). Although the editing site is distant from the interface between ChlN, our results indicate that the Pro substitution has a big impact on ChlN-ChlB complex formation. Consequently the Leu to Pro substitution in the α-helix likely has a significant impact on the overall tertiary structure that manifests a defect in this subunit interaction. Even though a single Arg substitution exerts only slight negative effects on the complex formation, our results also imply that the double substitution affects the activity of NB protein with an additive destabilization effect.

In a previous study using *L. decidua*, production of Chl was largely arrested after 7 days in cotyledons grown in the dark, despite maintenance of DPOR mRNA levels^11^. These authors also reported a significant decrease in the RNA editing efficiency of *chlB* mRNA from 7 to 14 days, suggesting a strong correlation between Chl production and RNA editing in dark-grown *L. decidua* seedlings. Our evaluation of ChlB variants in the present study indicates a causal relationship between RNA editing of *chlB* mRNA and DPOR activity.

Our results suggested that the DPOR activity is maintained at a high level by up-regulated transcription of the *chlLNB* genes and high efficient RNA editing under the dark conditions, and under the light conditions the DPOR activity decreases by down-regulated transcription and low efficiency of RNA editing. One consequence of RNA editing of the *chlB* mRNA may be to provide an additional regulatory layer of the DPOR activity in addition to the main transcriptional regulation in black pine chloroplasts.

It would be reasonable that the editing efficiency of *chlB* is higher in the dark, where DPOR is critical for Chl supply, than in the light in *P. thunbergii*. Under light conditions, LPOR is a better enzyme to reduce Pchlide than DPOR. LPOR, which is a small protein of approximately 30 kD, has a tolerance to oxygen and requires light and NADPH for its activity^31^. In contrast, DPOR is a relatively large protein complex (approximately 360 kD; consisting one NB protein and two L protein^30^) and requires reduced ferredoxin and ATP^8, 32^ with an oxygen-labile FeS cluster^25^. In addition, DPOR may compete for Pchlide with LPOR if both enzymes are present in the chloroplast. Given that the Pchlide binding site is located at the interface between ChlB and ChlN^14, 30^, the dissociation of NB protein may contribute to prevent competition for Pchlide between LPOR.

There are significant amounts of ChlB_PR left as a soluble protein in YFB14/NB2_PR strain, while soluble ChlN protein decreased obviously in the cell (Fig. 2B,c,d). This result implies that ChlB variant from pre-edited mRNA would be present as a homomeric protein in chloroplasts. Transgenic tobacco overexpressing pre-edited *chlB* of *P. thunbergii* in chloroplasts showed stimulation of root development, suggesting alternative functions of the ChlB variant derived from pre-edited mRNA^33^. Pre-edited mRNAs of *rps2* encoded by maize mitochondrial genome are also translated as well as edited *rps2* mRNA^34^. In animal nerve cells, proteins translated from pre-edited mRNA are likely to have different functions from those of corresponding proteins from edited mRNA^35^. If a homomeric ChlB protein translated from pre-edited mRNA also has a different function, RNA editing in *chlB* might work as a switch to change the function of ChlB in black pine. Further studies are needed to identify such additional functions of ChlB in photosynthetic organisms.

## Materials and Methods

### Cyanobacterial strains, mutants, and culture conditions

The cyanobacterial mutant YFB14 lacking *chlB* ^26^ was derived from *Leptolyngbya boryana* (formerly *Plectonema boryanum*) strain *dg5* ^36^, which was used as the host strain to overexpress *chlB* variants. YFB14 was cultivated in BG-11 medium with 15 µg ml^−1^ kanamycin under medium-intensity light conditions (40 µmol_photon_ m^−2^ s^−1^) using fluorescent lamps as described previously^16^. Transformants harboring the overexpression plasmids were cultivated as above in medium supplemented with 10 µg ml^−1^ chloramphenicol for plasmid maintenance. The medium was supplemented with glucose (30 mM) for heterotrophic growth in the dark.

### Plasmid construction

To introduce pre-editing amino acid substitutions into ChlB from *L. boryana*, pHANB2_P, pHANB2_R, and pHANB2_PR were constructed from pHANB2 (Supplementary Tables S2 and S3), which have *chlN–chlB* fragments in pASK-IBA5plus as the template^25^. The T-to-C substitution in the Leu209 codon (CTA) produced the Pro codon (CCA) and was introduced by PCR using primers PbNf1 and PbchlBedit-r1 (fragment II; Supplementary Table S2). The corresponding fragment without the substitution was amplified using primers PbNf1 and PbchlBWT-r1 (fragment I; Supplementary Table S2). The second mutation, which substituted the Trp216 codon (TGG) with the Arg codon (CGG), was introduced as a partial *chlB* fragment and was amplified using primers PbchlBedit-f1 and PbBr1 (fragment IV; Supplementary Table S2). The corresponding fragment without the substitution was amplified using primers PbchlBWT-f1 and PbBr1 (fragment III; Supplementary Table S2). Fragments I (II) and III (IV) have an identical 13-bp sequence that was used to overlap PCR^37^. The entire *chlN–chlB* fragment containing the L209P substitution (using fragments II and III), W216R substitution (using fragments I and IV), and both substitutions (using fragments II and IV) were then produced. These connected fragments were cloned into *Bsa*I sites in pASK-IBA5plus to yield pHANB2_P (L209P), pHANB2_R (W216R), and pHANB2_PR (both substitutions), respectively (Supplementary Table S3). Shuttle vectors expressing these *chlB* variants with *chlN* in *L. boryana* were constructed as described previously^25^. The *chlN–chlB* fragments were amplified by PCR using the primers PBHLI18-f1 and PBHchlNBr1 (Supplementary Table S2), with pHANB2_P, pHANB2_R, and pHANB2_PR as templates, and the PCR fragment was introduced into the *Sph*I-*Bam*HI sites of pPBHLI18^25^ to yield pHBNB2_P, pHANB2_R, and pHANB2_PR (Supplementary Table S3). These plasmids were then introduced into cyanobacterial cells by electroporation^38^.

### Determination of Chl and Pchlide

Pigments were extracted from the dark-grown cyanobacteria in 90% (v/v) methanol as described previously^16^. Chl and Pchlide contents in methanol extracts were determined by HPLC. For HPLC analyses, aliquots of methanol extracts were applied to a 4.6 × 150 mm Symmetry C8 3.5-µm column (Waters), and pigments were separated as described previously^39, 40^. Pchlide and Chl were eluted at 7.9 and 22.2 min, respectively, and their contents were determined using standard pigments.

### ChlB co-purification

Pre-edited mimic ChlB variants were co-purified with Strep-ChlN from soluble fractions of *L. boryana* ^25, 41^ and *E. coli* ^25, 41^ using a Strep-Tactin column as described previously^42^. The purification procedure was performed in an anaerobic chamber as described previously^16^. DPOR activity assay were then performed as described previously^24^.

### Western blot analysis

Crude and soluble fractions of *E. coli* were prepared as described previously^41^. Crude extracts of cyanobacterial cells were prepared and Western blot analyses were performed as described previously^16^. After SDS-PAGE, proteins were transferred onto PVDF membranes and were incubated with an antiserum against ChlB or ChlN from *L. boryana* ^15, 26^, followed by goat anti-rabbit IgG-horseradish peroxidase conjugate (Bio-Rad). Protein bands were visualized using a chemiluminescent substrate (ECL Western Blotting Analysis System; GE Healthcare).

### Pine cultivation and RNA extraction from cotyledons

Seedlings of the black pine *P. thunbergii* were grown under continuous light or continuous dark at 25 °C. Cotyledons were harvested at 10 and 14 days of age. RNA was extracted from cotyledons using RNeasy Plant Mini Kit (Qiagen) and DNase (RQ1 RNase-Free DNase; Promega) and was used for RT-PCR analyses and sub-cloning of *chlB* fragments. After preparation of cDNA from the extracted RNA, PCR was carried out using the gene-specific primers (Supplementary Table S2) with 25 cycles for *chlB*, *chlL*, *psbA*, *rbcL*, and *rrn23* genes and 30 cycles for *chlN* and *psbB* genes. Amplified *chlB* fragments were cloned into pGEM-T Easy Vector (Promega), and nucleotide sequences were determined in both directions using the primer pair M13 forward and M13 reverse (3730XI, ABI). For each condition 33 or 36 clones were sequenced (Supplementary Table S1). RNA editing efficiency was defined as the number of edited clones/total number of clones.

## Electronic supplementary material

Supplemental information
