# Supplementary material for: The Effect of Two Amino acid Residue Substitutions via RNA Editing on Dark-operative Protochlorophyllide Oxidoreductase in the Black Pine Chloroplasts
Source: Sci Rep. 2017 May 24;7:2377. doi: 10.1038/s41598-017-02630-2 (PMC5443842; doi:10.1038/s41598-017-02630-2)
Supplement: Supplementary file 1 — Supplemental information [file 41598_2017_2630_MOESM1_ESM.pdf]

Supplementary Information for

The effect of two amino acid residue substitutions via RNA editing on dark-operative protochlorophyllide oxidoreductase in the black pine chloroplasts

Haruki Yamamoto*, Junko Kusumi, Hisanori Yamakawa, and Yuichi Fujita

*To whom correspondence should be addressed: yamamoth@indiana.edu

This contains Supplementary Figure S1-S4 and Table S1-S3.

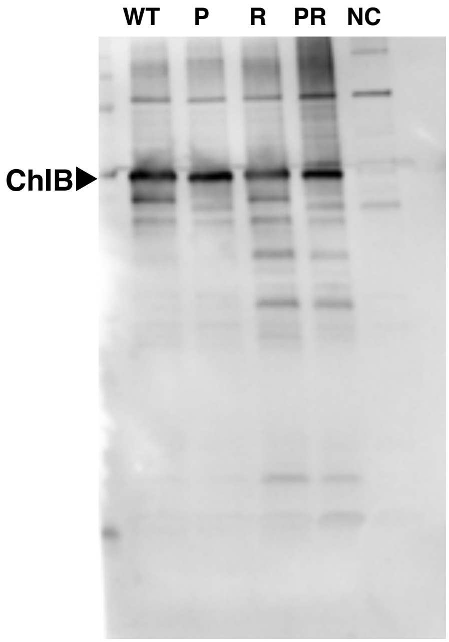

Figure S1 ChlB protein in the total extract of *L. boryana* strains.
Western blot analysis to show the content of ChlB proteins in total extract of YFB14/NB2 (WT), YFB14/NB2_P (P), YFB14/NB2_R (R), YFB14/NB2_PR (PR), and YFB14/H202 (NC) using anti-ChlB antiserum (3 μg protein/lane).

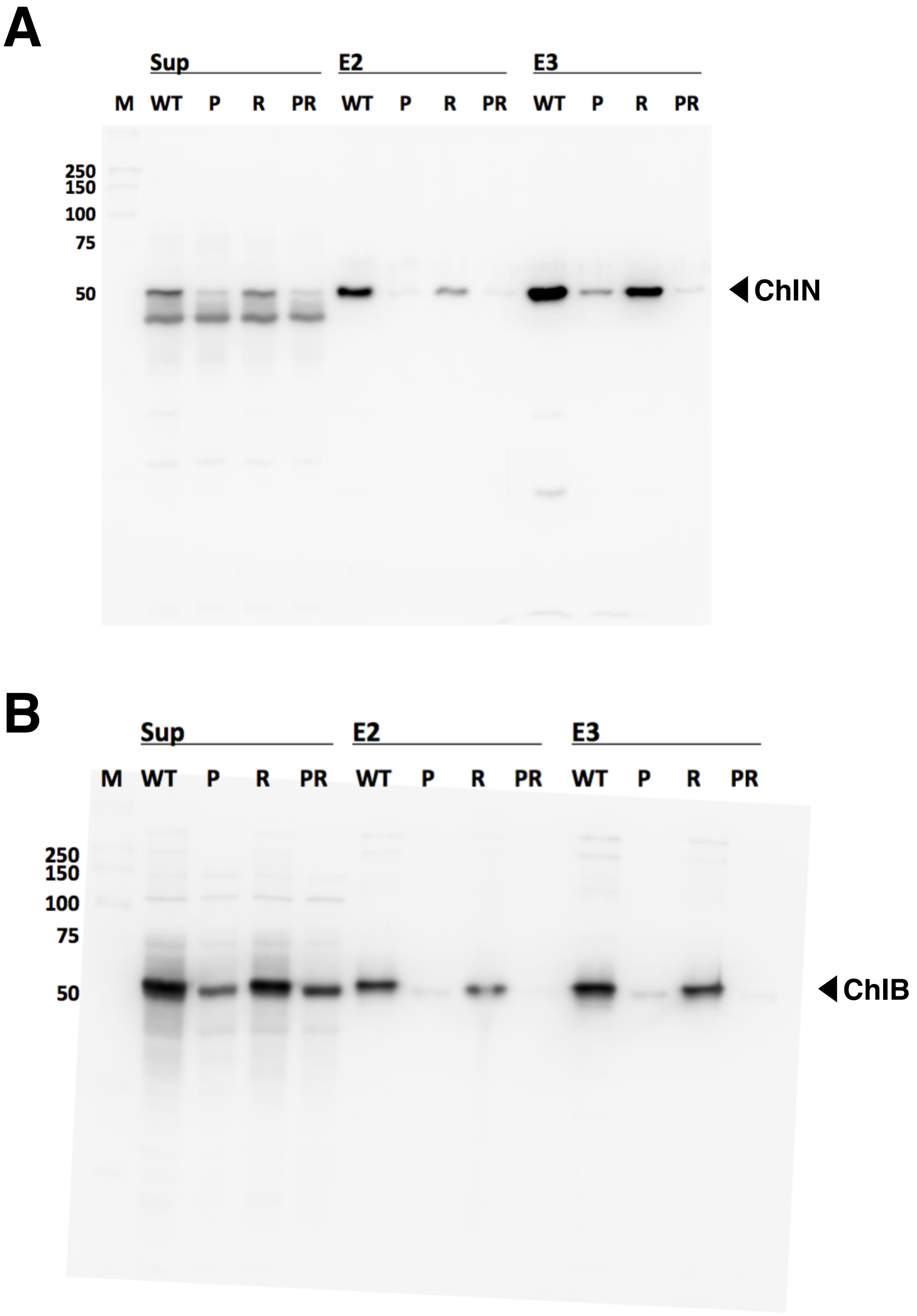

Figure S2 Co-purification of ChlB with Strep-ChlN from *L. boryana* soluble extracts

ChlN (A) and ChlB (B) proteins were detected in soluble fractions (Sup) using anti-ChlN and anti-ChlB antiserum, respectively. From the soluble fractions of YFB14/NB2 (WT), YFB14/NB2_P (P), YFB14/NB2_R (R), YFB14/NB2_PR (PR), Strep-ChlN was purified using the Strep-Tactin column and eluted 6 times. Second (E2) and third (E3) eluted fractions, which includes the most amount of protein, were applied to Western blot analysis.

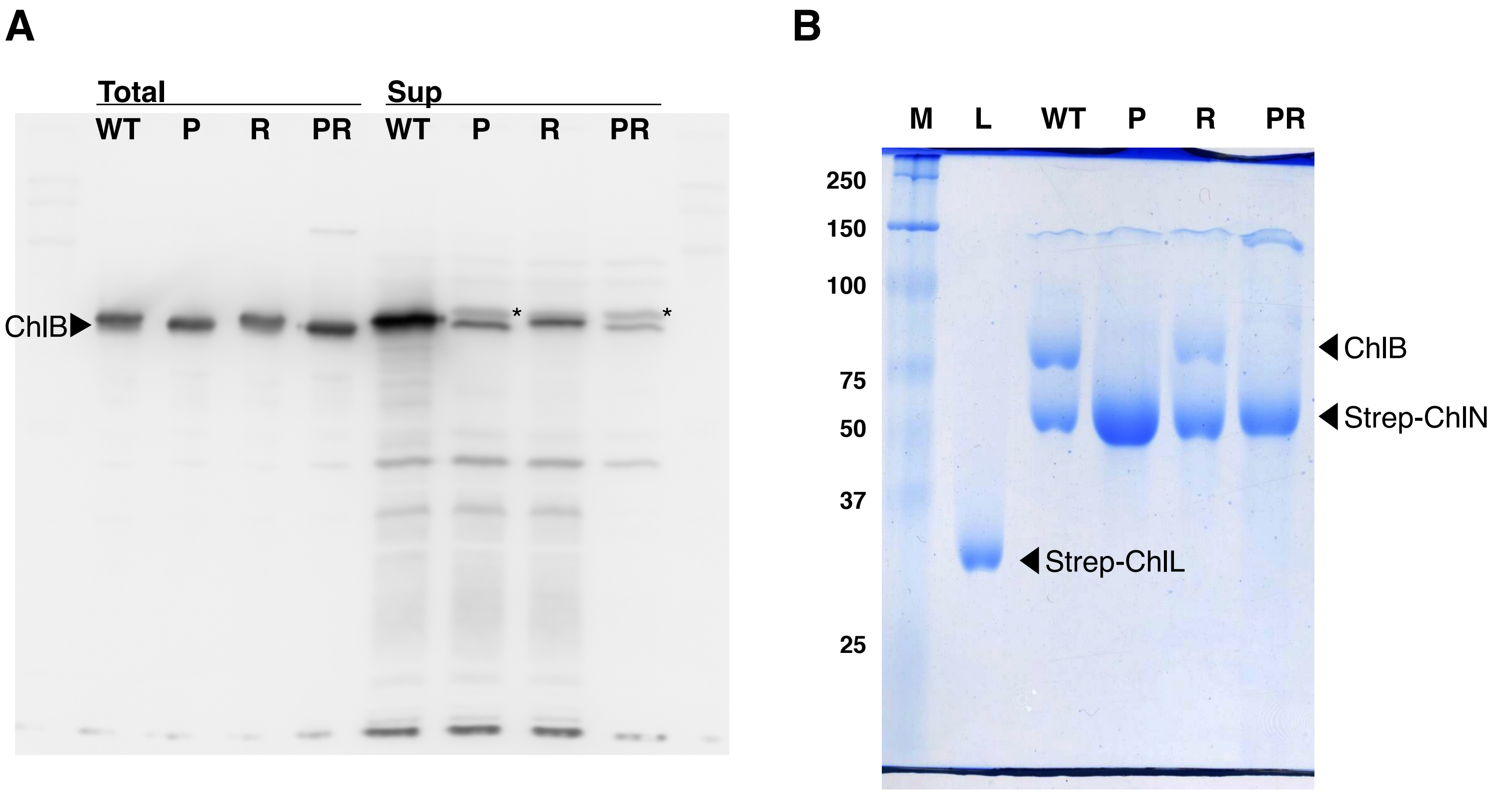

Figure S3 Co-purification of ChlB from *E. coli* extracts

(A) The amount of ChlB protein in the total (0.1 µg protein/lane) and soluble extracts (1.25 µg protein/lane) of BL21/pHANB2 (WT), BL21/pHANB2_P (P), BL21/pHANB2_R (R), and BL21/pHANB2_PR (PR) was checked by Western blot using anti-ChlB antiserum. A nonspecific signal detected just above the ChlB signal in the soluble fractions is shown by an asterisk. **(B)** SDS-PAGE profile of purified protein using Strep-Tactin column from the soluble extracts of BL21/pHANB2 (WT), BL21/pHANB2_P (P), BL21/pHANB2_R (R), and BL21/pHANB2_PR (PR). Strep-ChlL (L) was also purified from *L. boryana* for DPOR assay.

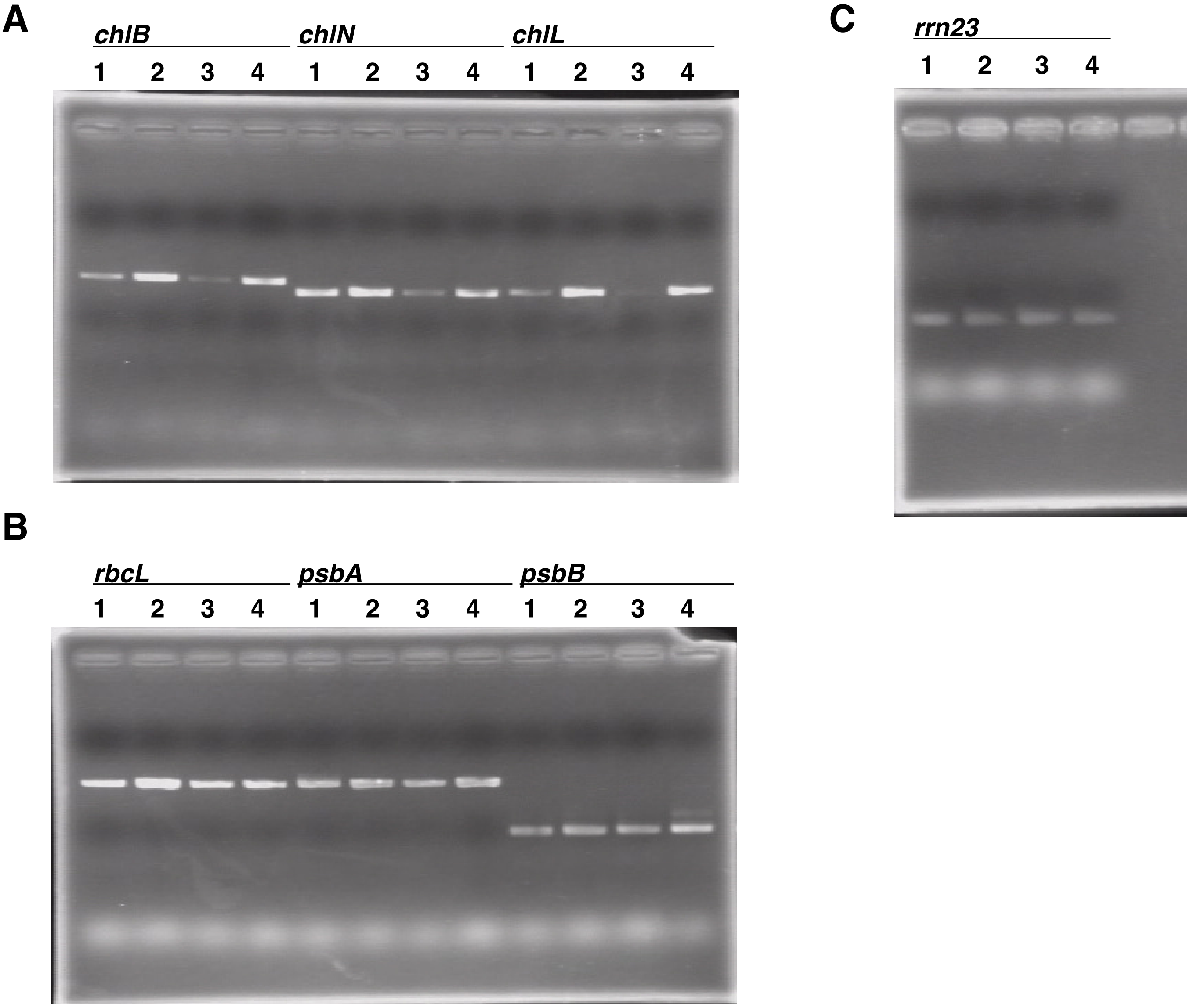

Figure S4 Semi-quantitive RT-PCR analysis

Semi-quantification of the transcripts of several chloroplast genes in cotyledons of seedlings grown in light (lanes 1 and 3) and dark (lanes 2 and 4) for 10 (lanes 1 and 2) and 14 days (lanes 3 and 4). **(A)** Three *chlB*, *chlN*, and *chlL* genes encode the DPOR subunits. **(B)** The *rbcL*, *psbA*, and *psbB* genes were treated as genes representative of photosynthesis in chloroplast genomes. **(C)** *rrn23* was used as an internal standard.

Supplementary Table S1 Number of sequenced cDNA clones of the *chlB* fragments

Sampling time (d)	Light (L) or dark (D)	Unedited	Singly edited		Completely edited	Total
		CCA/CGG	CCA/TGG	CTA/CGG	CTA/TGG	
10	L	9	2	1	21	33
	D	1	0	1	31	33
14	L	18	1	1	16	36
	D	3	2	1	30	36

Numbers of cDNA clones of unedited (CCA/CGG), singly edited (CTA/CGG, CCA/TGG) and completely edited (CTA/TGG) *chlB* fragments are shown.

Supplementary Table S2 Oligonucleotides used in this study

Primer name	Sequence of primer [a]	Use [b]	References
ChlB-F	5′-GATGTATGCTGGTCCTGCTC-3′	Forward primer for *chlB*	Kusumi et al. (2006)
PinusChlB-IR5.5	5′-ATAGGAGTTGTAGATACATAAGGCA-3′	Reverse primer for *chlB*	this study
ChlL-F	5′-ATAGCAGTTTACGGGAAAGG-3′	Forward primer for *chlL*	Kusumi et al. (2000)
ChlL-R	5′-TGAATAATTCCCGATCTGGA-3′	Reverse primer for *chlL*	Kusumi et al. (2000)
ChlN-IF	5′-GGAGAAGATACTGTGTTAGC-3′	Forward primer for *chlN*	Kusumi et al. (2006)
PinusChlN-rtR	5′-CATACCACATCTGATTAAGAATCT-3′	Reverse primer for *chlN*	this study
RbcL-F	5′-GTTTGGACCGATGGACTTAC-3′	Forward primer for *rbcL*	Kusumi et al. (2006)
RbcL-R	5′-ACACCTGGCATAGATACCC-3′	Reverse primer for *rbcL*	Kusumi et al. (2006)
PsbA-F	5′-TGGTTCGGCGTCTTGATGAT-3′	Forward primer for *psbA*	this study
PsbA-R	5′-GGAAAGTTGAGAGCATTACG-3′	Reverse primer for *psbA*	this study
PsbB-F	5′-TGACAGGGTCAGTTTTGGTG-3′	Forward primer for *psbB*	this study
PsbB-R	5′-TCTCCCAGTTTTTGGAATGC-3′	Reverse primer for *psbB*	this study
23SRTf	5′-AATGAGCCGGCGACTTATAGG-3′	Forward primer for *rrn23*	Breznenová et al. (2010)
23SRTr	5′-GGGTCCATAAGCAGTGACAATTG-3′	Reverse primer for *rrn23*	Breznenová et al. (2010)
PbNf1	5′-GTCTGAGGTCTCAGCGCCACTCTCGCTGATGCTCAACC-3′	Forward primer for pHANB2_P, R, PR (*Bsa*I)	this study
PbchlBWT-r1	5′-CCGAGGAAGGGATTTTAGTTCATGAACACTTGCG-3′	Reverse primer for fragment I	this study
PbchlBedit-r1	5′-CCGAGGAAGGGATTTTGGTTCATGAACACTTGCG-3′	Reverse primer for fragment II	this study
PbchlBWT-f1	5′-ATCCCTTCCTCGGGCTTGGTTTAACCTGGTGCC-3′	Forward primer for fragment III	this study
PbchlBedit-f1	5′-ATCCCTTCCTCGGGCTCGGTTTAACCTGGTGCC-3′	Forward primer for fragment IV	this study
PbBr1	5′-GTCATAGGTCTCATATCCTAAGCACCCACTGCTTC-3′	Reverse primer for pHANB2_P, R, PR (*Bsa*I)	this study
PBHLI18-f1	5′-GATCGGCATGCTGGCTAGCTGGAGCCACCCGCAGTTCG-3′	Forward primer for pHBNB2_ P, R, PR (*Sph*I)	Yamamoto et al. (2009)
PBHchlNBr1	5′-ATAGGATCCCTAAGCACCCACTGCTTC-3′	Reverse primer for pHBNB2_ P, R, PR (*Bam*HI)	Yamamoto et al. (2009)

[a] Introduced restriction site are underlined and the complementary sequences for overlapping PCR are double underlined. The nucleotides used to prepare pre-edited mimic *chlB* are shown by dots.

[b] Introduced restriction sites are shown in parentheses.

Supplementary Table S3 Plasmids used in this study [a]

Plasmid	Description	Source or reference
pASK-IBA5plus		IBA
pHANB2	*chlN–chlB* in *Bsa*I sites of pASK-IBA5plus	Yamamoto et al. (2009)
pHANB2_P	*chlN–chlB* (L209P) in *Bsa*I sites of pASK-IBA5plus	this work
pHANB2_R	*chlN–chlB* (W216R) in *Bsa*I sites of pASK-IBA5plus	this work
pHANB2_PR	*chlN–chlB* (L209P, W216R) in pASK-IBA5plus	this work
pPBH202	Shuttle vector between *E. coli* and *L. boryana*	Yamamoto et al. (2009)
pPBHLI18	Shuttle vector expressing *chlL* as His-tag fusion protein	Yamamoto et al. (2009)
pHBNB2	A PCR-amplified *strep-chlN–chlB* in *Sph*I and *Bam*HI sites of pPBHLI18	Yamamoto et al. (2009)
pHBNB2_P	A PCR-amplified *strep-chlN–chlB* from pHANB2_P in *Sph*I and *Bam*HI sites of pPBHLI18	this work
pHBNB2_R	A PCR-amplified *strep-chlN–chlB* from pHANB2_R in *Sph*I and *Bam*HI sites of pPBHLI18	this work
pHBNB2_PR	A PCR-amplified *strep-chlN–chlB* from pHANB2_PR in *Sph*I and *Bam*HI sites of pPBHLI18	this work

[a]All genes encoding DPOR subunits are derived from *L. boryana*.
